# Supplementary material for: Streams of conscious visual experience
Source: Commun Biol. 2024 Jul 27;7:908. doi: 10.1038/s42003-024-06593-9 (PMC11283449; doi:10.1038/s42003-024-06593-9)
Supplement: Supplementary file 2 — Reporting summary [file 42003_2024_6593_MOESM2_ESM.pdf]

nature portfolio

Corresponding author(s): Mar Martín-Signes

Last updated by author(s): Jun 21, 2023

Reporting Summary

Nature Portfolio wishes to improve the reproducibility of the work that we publish. This form provides structure for consistency and transparency in reporting. For further information on Nature Portfolio policies, see our Editorial Policies and the Editorial Policy Checklist.

Statistics

For all statistical analyses, confirm that the following items are present in the figure legend, table legend, main text, or Methods section.

n/a	Confirmed	
☐	☒	The exact sample size (*n*) for each experimental group/condition, given as a discrete number and unit of measurement
☒	☐	A statement on whether measurements were taken from distinct samples or whether the same sample was measured repeatedly
☐	☒	The statistical test(s) used AND whether they are one- or two-sided<br>*Only common tests should be described solely by name; describe more complex techniques in the Methods section.*
☒	☐	A description of all covariates tested
☐	☒	A description of any assumptions or corrections, such as tests of normality and adjustment for multiple comparisons
☒	☐	A full description of the statistical parameters including central tendency (e.g. means) or other basic estimates (e.g. regression coefficient) AND variation (e.g. standard deviation) or associated estimates of uncertainty (e.g. confidence intervals)
☐	☒	For null hypothesis testing, the test statistic (e.g. *F*, *t*, *r*) with confidence intervals, effect sizes, degrees of freedom and *P* value noted<br>*Give P values as exact values whenever suitable.*
☒	☐	For Bayesian analysis, information on the choice of priors and Markov chain Monte Carlo settings
☒	☐	For hierarchical and complex designs, identification of the appropriate level for tests and full reporting of outcomes
☒	☐	Estimates of effect sizes (e.g. Cohen's *d*, Pearson's *r*), indicating how they were calculated

Our web collection on statistics for biologists contains articles on many of the points above.

Software and code

Policy information about availability of computer code

Data collection	In this study, three datasets previously collected and published were reanalyzed. In the original studies, data was collected using E-Prime and a Tesla Siemens TRIO MRI scanner.
Data analysis	FEAT (FSL, FMRIB's Software Library, Woolrich et al., 2001).

For manuscripts utilizing custom algorithms or software that are central to the research but not yet described in published literature, software must be made available to editors and reviewers. We strongly encourage code deposition in a community repository (e.g. GitHub). See the Nature Portfolio guidelines for submitting code & software for further information.

Data

Policy information about availability of data

All manuscripts must include a data availability statement. This statement should provide the following information, where applicable:

- Accession codes, unique identifiers, or web links for publicly available datasets
- A description of any restrictions on data availability
- For clinical datasets or third party data, please ensure that the statement adheres to our policy

fMRI results maps are publicly available via Neurovault (https://neurovault.org/collections/15553/). The conditions of the ethics approvals of the datasets do not permit public archiving or sharing of anonymized raw study data.

Research involving human participants, their data, or biological material

Policy information about studies with human participants or human data. See also policy information about sex, gender (identity/presentation), and sexual orientation and race, ethnicity and racism.

Reporting on sex and gender	In this study, three datasets previously collected and published were reanalyzed. In the original studies, participants were asked about their sex and in all samples there are male and female participants. In this study, this variable was not analyzed.
Reporting on race, ethnicity, or other socially relevant groupings	In this study, three datasets previously collected and published were reanalyzed. In the original studies, these variables were not collected.
Population characteristics	In this study, three datasets previously collected and published were reanalyzed. In the original studies, age was collected and can be checked in the original publications.
Recruitment	In this study, three datasets previously collected and published were reanalyzed. In the original studies, this information was not stated but participants were recruited through advertisements and emails.
Ethics oversight	Ethics Committee of the INSERM (France) and the University of Granada (Spain).

Note that full information on the approval of the study protocol must also be provided in the manuscript.

Field-specific reporting

Please select the one below that is the best fit for your research. If you are not sure, read the appropriate sections before making your selection.

☐ Life sciences ☒ Behavioural & social sciences ☐ Ecological, evolutionary & environmental sciences

For a reference copy of the document with all sections, see nature.com/documents/nr-reporting-summary-flat.pdf

Behavioural & social sciences study design

All studies must disclose on these points even when the disclosure is negative.

Study description	Quantitative experimental.
Research sample	Reanalysis of data from 3 datasets (Chica, 2013, 2016, Martín-Signes, 2019).
Sampling strategy	Data reanalyzed in this study came from 3 datasets (Chica, 2013, 2016, Martín-Signes, 2019) in which no calculation of sample size was included but where significant effects were obtained. The sampling procedure in these studies was convenience.
Data collection	In this study, three datasets previously collected and published were reanalyzed. In the original studies, data was collected using E-Prime and a Tesla Siemens TRIO MRI scanner. During data collection, participants were by themselves in the scanner, while the technician and the researcher were in the control room. Experimental conditions were presented randomly, without the control of the researcher.
Timing	Data reanalyzed in this study came from 3 datasets (Chica, 2013, 2016, Martín-Signes, 2019) in which this information was not stated.
Data exclusions	In this study, three datasets previously collected and published were reanalyzed. In the original studies, 3 participants were excluded due to technical difficulties during fMRI data acquisition or excessive head motion during imaging (this criterion was already established based on voxel dimensions).
Non-participation	Data reanalyzed in this study came from 3 datasets (Chica, 2013, 2016, Martín-Signes, 2019) in which this information was not stated.
Randomization	Experimental conditions were presented randomly to each participant.

Reporting for specific materials, systems and methods

We require information from authors about some types of materials, experimental systems and methods used in many studies. Here, indicate whether each material, system or method listed is relevant to your study. If you are not sure if a list item applies to your research, read the appropriate section before selecting a response.

Materials & experimental systems

n/a	Involved in the study
☒	☐ Antibodies
☒	☐ Eukaryotic cell lines
☒	☐ Palaeontology and archaeology
☒	☐ Animals and other organisms
☒	☐ Clinical data
☒	☐ Dual use research of concern
☒	☐ Plants

Methods

n/a	Involved in the study
☒	☐ ChIP-seq
☒	☐ Flow cytometry
☐	☒ MRI-based neuroimaging

Magnetic resonance imaging

Experimental design

Design type: Task, event-related.

Design specifications: Dataset 1 (Chica, 2016): The experiment consisted of two sessions with 5 functional scans of 12 min duration each (920 trials in total). Trial duration: 6000 ms.
Dataset 2 (Chica, 2013): The experiment consisted of one session with 5 functional scans of 7 min duration (280 trials in total). Trial duration: 6000 ms.
Dataset 3 (Martín-Signes, 2019): The experiment consisted of 2 sessions with 5 functional scans of 8 min duration each (600 trials in total). Trial duration: 6000 ms.

Behavioral performance measures: Reaction time, percentage of seen targets, and correct target discrimination were collected.

Acquisition

Imaging type(s): Structural, functional.

Field strength: 3

Sequence & imaging parameters: Full descriptions of the acquisition parameters can be found in the original publications (Chica et al., 2013, 2016; Martín-Signes et al., 2019). Whole-brain fMRI was conducted on two different 3 Tesla Siemens TRIO MRI scanners using a whole-head coil. Functional images were acquired using a gradient-echo echo-planar pulse sequence with the following parameters for the alerting task (time-to-repetition [TR] = 2000 ms, time-to-echo [TE] = 25 ms, 39 axial 3-mm cubic slides, no inter-slice gap, flip angle = 75°, field of view [FoV] = 220 mm, 372 volumes acquired per run), the orienting task (TR = 2000 ms, TE = 25 ms, 34 axial 2.5 x 2.5 x 3-mm slides, no inter-slice gap, flip angle = 75°, FoV = 220 mm, 220 volumes acquired per run), and the executive attention task (TR = 2000 ms, TE = 25 ms, 35 3.4-mm cubic axial slides, no inter-slice gap, flip angle = 75°, FOV = 220 mm, 245 volumes per run). High-resolution T1-weighted anatomical images were also collected.

Area of acquisition: whole brain

Diffusion MRI: ☐ Used ☒ Not used

Preprocessing

Preprocessing software: Preprocessing routines and analyses were performed using FEAT (FSL, FMRIB's Software Library, Woolrich et al., 2001).

Normalization: Structural and functional volumes of each participant were coregistered using the Boundary-Based Registration function. Next, the structural volume was registered to a standard image and a similar transformation was applied to the functional volume using a non-linear registration with 12 degrees of freedom. During normalization, volumes were sampled to 2 mm isotropic voxels and standard images were based on the MNI152 stereotaxic space.

Normalization template: MNI152

Noise and artifact removal: Images were corrected for differences in timing of slice acquisition and were realigned to the middle volume by means of rigid-body transformation for motion correction using MCFLIRT (Jenkinson et al., 2002). Motion plots were visually inspected to discard those runs with excessive motion (i.e., relative motion > than half of voxel size, or absolute motion > than voxel size). A 128s high-pass filter was used to eliminate contamination from slow drift of signals.

Volume censoring: Outlier scans corrupted by large motion were detected using the tool fsl_motion_outliers and regressed out. The number of outlier scans never exceeded 20% of the total scans in a run.

Statistical modeling & inference

Model type and settings	Intra-subject brain activations of the contrast of interest were calculated using fixed effects. Group effects were carried out using FLAME 1.
Effect(s) tested	The contrast of interest was Seen > Unseen targets. We also performed a conjunction analysis between the result maps of the three experiments.

Specify type of analysis: ☒ Whole brain ☐ ROI-based ☐ Both

Statistic type for inference (See Eklund et al. 2016)	Cluster-wise. Z-threshold of >2.3 and a corrected cluster significance threshold of p=0.05.
Correction	TFCE (Threshold-Free Cluster Enhancement)

Models & analysis

n/a	Involved in the study
☒	☐ Functional and/or effective connectivity
☒	☐ Graph analysis
☒	☐ Multivariate modeling or predictive analysis
